# ADHD in adults and relationship with executive functioning and quality of life

**DOI:** 10.1192/j.eurpsy.2025.1872

**Published:** 2025-08-26

**Authors:** I. M. V. Siqueira, L. R. R. Carreiro, N. M. Dias, J. C. M. O. B. Delorenzi, S. M. Blascovi-Assis, A. G. Seabra

**Affiliations:** 1 Mackenzie Presbyterian University, Sao Paulo; 2 Federal University of Santa Catarina, Florianopolis, Brazil

## Abstract

**Introduction:**

Attention-deficit/hyperactivity disorder (ADHD) is a neurodevelopmental disorder characterized by inattention, disorganization, and/or hyperactivity-impulsivity. It is associated with cognitive deficits and functional impairments. A frequent cognitive impairment, likely related to hypofunctioning of the prefrontal cortex and its connections, involves executive functions, a set of skills related to self-control and goal-directed activities. The disorder also significantly affects quality of life, being associated, for example, with higher unemployment, relationship problems, and lower average income compared to individuals without the disorder.

**Objectives:**

To investigate the performance in executive functions and quality of life in individuals with ADHD compared to a control group, and to relate these with the severity of symptoms.

**Methods:**

Forty individuals aged 18-50 participated, including 10 with an ADHD diagnosis and 30 controls matched for sex, age, and education in a case-control design (3 controls per case). Measures included: a) the Adult Self-Report Scale (ASRS) with 18 items, based on the DSM criteria for ADHD (total score used); b) the computerized Stroop Test (Victoria version) to assess executive functions, with three phases: reading words in black (control 1), naming colors in circles (control 2), and naming colors in words while inhibiting reading (incongruent phase). Reaction times for each part and interference times (part 3 minus part 2) were calculated; c) the WHOQOL questionnaire, assessing quality of life across four domains (physical, psychological, social relationships, and environment), with domain scores used. Given data normality, Student’s t-tests compared group means.

**Results:**

ADHD participants had significantly poorer executive functions, with longer reaction times in the Stroop Test part 3 (M = 1.05; SD = .63) compared to controls (M = .69; SD = .32), and higher interference reaction times (M = .28; SD = .34 for ADHD) compared to controls (M = .10; SD = .16). ADHD individuals also reported lower quality of life in social relationships (WHOQOL Domain 3) (M = 3.3, SD = .76) compared to controls (M = 3.8; SD = .65). Significant correlations indicated that more ASRS symptoms were associated with longer reaction times in Stroop Test parts 2 and 3, higher interference reaction times, and lower quality of life in social relationships.

**Conclusions:**

The study highlights deficits in executive functions, particularly inhibition in incongruent situations, and reduced quality of life, especially in social relationships, in individuals with ADHD. These impairments correlate with the severity of symptoms, emphasizing the need to address executive function disturbances and quality of life in ADHD treatment.

**Disclosure of Interest:**

None Declared

